# Omics Evidence Chains for Complex Traits in Beef Cattle: From Cross-Layer Colocalization to Genetic Evaluation and Application

**DOI:** 10.3390/biology14121725

**Published:** 2025-12-01

**Authors:** Ying Lu, Dongfang Li, Ruoshan Ma, Yuyang Gao, Zhendong Gao, Yuwei Qian, Dongmei Xi, Weidong Deng, Jiao Wu

**Affiliations:** 1 Yunnan Provincial Key Laboratory of Animal Nutrition and Feed, Faculty of Animal Science and Technology, Yunnan Agricultural University, Kunming 650201, China; yinglu_1998@163.com (Y.L.); dfli0927@163.com (D.L.);; 2 State Key Laboratory of Conservation and Utilization of Bio-Resources in Yunnan, Kunming 650201, China

**Keywords:** multi-omics, colocalization, causal modeling, reaction norms, genetic evaluation

## Abstract

Multi-omics studies have multiplied associations, but many still lack causal resolution and a clear path to application. We present a practical roadmap built on four sequential steps: first, identify signals from genome-wide association studies; second, confirm these signals through regulatory colocalization and transcriptome-wide association analyses; third, integrate the evidence using network analyses and causal inference; and, fourth, test shortlisted candidates through functional and phenotypic validation. The roadmap is supported by three safeguards that make results reliable and reusable: containerized workflows that ensure end-to-end reproducibility, harmonization across batches with concise minimum-information records, and consistent identifier mapping with quality control across data layers. Across four classes of traits—growth and development, carcass and meat quality, reproduction, and environmental adaptation and resilience—we prioritize signals that remain robust across ancestries and environments, highlight modules with explicit regulatory support, and advance candidates that have already progressed to functional testing. Two application tracks follow from this process: integrating stable candidates into selection indices with context-dependent weighting, and recording and targeting mechanistic nodes for nutritional and management interventions. Taken together, this roadmap improves causal interpretability, strengthens cross-population robustness, and shortens the path from statistical association to genetic evaluation and industry uptake.

## Simple Summary

Beef cattle breeding has entered the multi-omics era, where data from the genome, transcriptome, proteome, and metabolome can be combined to reveal how genes influence complex traits such as growth, meat quality, reproduction, and environmental adaptation. However, many associations discovered in large-scale studies remain statistical and lack direct biological meaning. In this review, we summarize a practical roadmap that links genomic signals to causal genes and then to breeding applications. We outline how to integrate evidence through cross-layer colocalization, network and causal inference, and functional validation, while maintaining reproducibility and cross-population comparability. By organizing dispersed omics information into a traceable evidence chain, this framework helps to identify stable and biologically credible markers that can be incorporated into selection indices and management programs. Ultimately, it shortens the path from data discovery to genetic improvement, providing a systematic strategy for translating multi-omics discoveries into actionable decisions in beef cattle breeding.

## 1. Introduction

As one of humanity’s most important domesticated species, cattle descended from the *Eurasian aurochs* (*Bos primigenius*) and underwent at least two independent domestication events in the early Holocene: Humpless cattle in the Near East (Fertile Crescent; *Bos taurus*, ~10,000 years ago) and zebu in South Asia (*Bos indicus*, ~8000 years ago). The possible third domestication event in North Africa remains debated [1]. Later, prehistoric migrations and trade facilitated admixture between the two lineages across Eurasia and Africa, producing composite ancestries [2]. Some East Asian breeds additionally show structural variations and introgression from wild populations [3]. At the industry level, the annual production of beef and buffalo meat has stabilized at approximately 80 million metric tons in recent years. Over the next decade, global beef supply–demand is expected to remain broadly steady, with moderate growth in middle-income regions [4]. These origins and evolutionary processes have shaped the standing genetic diversity and adaptive potential of modern breeds, providing biological and industry foundations for deploying multiple-omics (multi-omics) evidence to guide regionally tailored selection and environment–breed matching [1,2].

However, despite remarkable progress in genomic selection, most associations discovered in large-scale studies remain statistical and lack direct biological interpretability, which limits their practical incorporation into breeding indices. In addition, inconsistent data standards and batch effects still constrain cross-population comparability and reuse. To address these challenges, we propose an evidence-chain framework that organizes multi-omics information—from genome to transcriptome, epigenome, and metabolome—into a reproducible and causally interpretable pathway linking genomic signals to functional mechanisms and breeding applications.

Multi-omics refers to the joint acquisition and integration of multiple molecular phenotypes within a single study system—spanning the genome, epigenome, transcriptome (including splicing), cis-regulatory layers, proteome, metabolome, and trait-proximal phenotypes and production records (phenomics) [5,6]. Methodologically, common integration strategies include early, intermediate, and late integration, as well as hybrid frameworks: either aligning features first and modeling jointly, or modeling separately and then merging at an evidence layer; these approaches are often paired with colocalization and causal inference to maximize cross-layer consistency and interpretability [5,7,8,9]. In livestock breeding and production, the introduction of systems genomics and multi-omics is helping to close the loop from marker associations to functional mechanisms and, ultimately, to selectable phenotypic improvement for complex traits in growth, meat production, reproduction, and stress adaptation [10,11].

The crux of multi-omics is not information volume but the organization of dispersed evidence into a traceable causal chain. For complex traits in beef cattle, a single genome-wide association study (GWAS) signal is rarely directly translatable. Candidate loci achieve verifiable, actionable status only when genomic associations show spatiotemporally specific support from expression and regulatory layers, proteomic and metabolomic data, and coherent explanation within network- and causality-based frameworks [9,12]. Compared with existing reviews in the past two years that have summarized general progress in livestock multi-omics or highlighted specific data layers such as genomics, proteomics, transcriptomics, or metabolomics [12,13,14], our review differs in both focus and structure. Specifically, we emphasize an integrative and methodological framework that links multi-omics evidence to causal inference and its translation into genetic evaluation, which has not been systematically addressed in recent reviews. Accordingly, we adopt a methodological layering approach rather than trait-by-trait enumeration. First, we discuss strengthening evidence from association to colocalization. Next, we introduce network- and causality-based frameworks to reduce confounding. Finally, we emphasize validation and translation in experimental and breeding contexts.

In parallel, we emphasize batch-effect control and workflow reproducibility to ensure comparability and cumulative progress across populations and platforms. As a methodological review centered on complex traits in beef cattle, we outline an evidence chain from association, to colocalization, to network- and causality-based frameworks, and finally to validation and translation, specifying where each link interfaces with selection-index integration. Practitioners can use this roadmap to build a minimal, reproducible workflow from signals to decisions and to select fit-for-purpose evidence and phenotypic anchors aligned with production goals. Importantly, this roadmap is portable across beef cattle and other animals, providing a unified interface for selection-index integration and reaction-norm modeling.

## 2. Single-Omics Evidence and Trait Dissection Progress from Association to Prioritized Candidates

Genes shape complex traits not only through effects on protein structure and metabolic pathways, but also—often predominantly—through cis- and trans-regulatory networks that control gene expression, splicing, and epigenetic state [15,16]. In single-omics analyses, evidence typically accumulates along a stepwise chain: genome-wide association identifies statistical loci, regulatory colocalization and transcriptome-wide association refine these loci to expression-linked variants, network and causal analyses constrain potential drivers, and functional perturbation assays validate causal elements [15,17,18,19,20,21]. This progression—from variant to gene or regulatory element, to pathway, and ultimately to phenotype—provides a framework for developing testable hypotheses and practical checkpoints for breeding decisions [22].

Evidence from expression, splicing, and epigenomic layers at the same locus is combined to orient gene function, while cross-omics integration and formal causal inference are presented in the subsequent section.

### 2.1. Discovery-Stage Signals for Growth and Development

For growth and development, production and genetic evaluation center on output and efficiency: body size and longitudinal growth metrics (body weight, height/length, heart girth, birth/weaning/yearling weights, average daily gain), together with carcass yield traits (e.g., hot carcass weight, ribeye area, backfat thickness) and feed-efficiency indicators—including feed conversion ratio (FCR), residual feed intake (RFI), and residual intake and gain (RIG)—which jointly define the energetic and metabolic basis of performance [23,24,25]. In most production systems, these traits carry explicit economic weights and consistently drive finishing efficiency and grading/pricing across supply chains [26,27]. Standardized recording and estimated breeding value (EBV)/index frameworks, supported by ultrasound/harvest data, enable population-scale gain with measurable responses [28,29].

For body size, weight, and carcass traits, we structure evidence as an exemplar–pattern–gate progression focused on a few anchors and concise criteria. As the exemplar, the *PLAG1-LCORL*/*NCAPG* region serves as a cross-ancestry anchor: establish association stability with GWAS/meta-GWAS, then test tissue-consistent expression quantitative trait loci (eQTL)/transcriptome-wide association study (TWAS)/sQTL in skeletal muscle/bone. When high colocalization probability aligns with tissue-specific expression, a local cis-regulatory mechanism is most parsimonious [22,30].

Beyond this anchor, organize residual signals into growth modules—*IMPAD1*, *PENK*, *STC2*, *CPEB4* for myogenesis/chondrogenesis, and *CSMD3*, *LAP3*, *SYN3*, *FAM19A5*, *TIMP3* for extracellular matrix (ECM) remodeling—while overlaying selective-sweep/extended-haplotype profiles to separate conserved from lineage-enriched effects [12,16,31,32,33,34].

For translation, apply a compact gate: cross-population replication plus colocalization/mediation support, evaluated within network context; functional readouts confirm direction and scale. Implementation details—such as prior weighting and deployment—are developed later within the multi-omics framework.

### 2.2. Discovery-Stage Signals for Carcass and Meat Quality

Carcass and meat-quality traits center on fat deposition and increase intramuscular fat (IMF) distribution; key indicators include subcutaneous backfat thickness, IMF content/marbling, and fatty-acid profile (monounsaturated fatty acid (MUFA)/saturated fatty acid (SFA), C18:1, desaturation index) [35], coupled with tenderness/Warner–Bratzler shear force, juiciness, and flavor [23]. On the market side, IMF and lipid profile drive grading and price premiums; on the improvement side, these traits are implemented in EBVs/indices, while GWAS/TWAS and integrative omics sharpen locus resolution [36,37,38].

For IMF and fat distribution, we advocate a module-then-locus-then-prioritization workflow. In the longissimus dorsi and adipose tissue, two recurrent axes—lipid-droplet biogenesis and fatty-acid transport/desaturation chemistry—are repeatedly enriched in pathway analyses, with a canonical *FABP4*/*SCD*/*ADIRF* backbone [37,38]. Rigorous GWAS–TWAS colocalization should be used to narrow broad associations to experimentally tractable candidates, and studies are encouraged to report explicit colocalization probabilities or TWAS-based mediation estimates to strengthen causal inference [37,38]. Candidate prioritization is then driven by the joint consideration of network centrality and measurable phenotypes: genes whose effects align with IMF percentage, fatty-acid profile, and instrument-based readouts such as shear force or near-infrared spectroscopy should be advanced to functional validation first [36,39].

To identify robust, selection-relevant loci, phenotype multiple cohorts under contrasted diet regimens to capture population and nutrition dependencies, and treat the desaturation index and the full-spectrum fatty-acid profile as mediators for pathway-level testing—particularly to position *SCD* and *FABP4* within a defensible causal chain [40,41,42,43,44]. This study design enriches signals that replicate across environments and that are mechanistically consistent with lipid handling, thereby increasing the probability that validated targets translate into improvements in EBVs and selection indices.

### 2.3. Discovery-Stage Signals for Reproductive Traits

Reproductive performance in beef cattle encompasses female puberty onset (age at puberty, APU), age at first calving (AFC), conception and pregnancy metrics (heifer conception rate, HCR; heifer pregnancy, HP; cow conception rate, CCR; days to calving; calving interval, CI), sustained fertility (stayability), and male indicators including scrotal circumference (SC), semen quality, and fertilizing capacity [45]. These traits determine calving rates, open days, and culling risk and thus are primary drivers of herd profitability; nevertheless, their typically low heritabilities and high sensitivity to environment and nutrition underscore the need for large-scale, standardized phenotyping and integrative omics to improve dissection and selection efficiency [23]. Recent work combining GWAS/TWAS with colocalization and single-cell plus epigenomic profiling is progressively translating multilayer signals—spanning axis-level neuroendocrine control, the gonadal microenvironment, and gametogenesis—into actionable molecular targets and selection indicators [9,46,47].

Along the hypothalamus–pituitary–gonadal (HPG) axis, transcriptomic and co-expression analyses consistently implicate neuropeptide/endocrine nodes such as *POMC*, *CHGA*, and *PENK* in the initiation of puberty [46], with foundational evidence from human/animal neuroendocrine studies [8,48]. For candidates expressed in the hypothalamus and pituitary, studies should report TWAS [20,21] and colocalization [19] evidence together with network centrality/module membership (weighted gene co-expression network analysis (WGCNA) [15] to strengthen causal interpretation, while explicitly modeling energy balance and nutritional covariates to minimize management/nutrition confounding [49]. Shifting to the ovarian/uterine niche, m6A RNA modification, alternative splicing, and noncoding RNAs jointly orchestrate folliculogenesis and implantation—covering pathways such as BMP15/BMP6/HOMER1/WNT signaling—with an *ALKBH5*–*BMP15* m6A mechanism in cattle recently linked to puberty timing [47] and aligning with single-cell atlases of early embryonic lineage specification [50]. To convert hierarchical signals into testable loci, jointly localize m6A-CLIP/MeRIP-seq peaks with sQTLs in granulosa cells and cumulus–oocyte complexes, complemented by cell type-weighted expression from spatial/single-cell data [51] to confirm lineage assignment, and by causal perturbation of candidate cis-regulatory elements using clustered regularly interspaced short palindromic repeats interference/activation (CRISPRi/a) or enhancer-targeting approaches [17,18,52].

On the male side, stage specificity across spermatogonial, primary spermatocyte, and round spermatid transitions is shaped by circular RNA (circRNA)-mediated buffering and chromatin accessibility. Combining single-cell RNA sequencing (scRNA-seq) with single-cell assay for transposase-accessible chromatin using sequencing (scATAC-seq)/cleavage under targets and tagmentation (CUT&Tag) delineates stage-specific open chromatin and transcription-factor occupancy [53,54,55], while siRNA/ASO-based perturbation of focal circRNAs provides functional readouts of regulatory cascades [9,56,57]. Recent single-cell and m6A-epitranscriptomic analyses have identified *ALKBH5*-mediated methylation of *BMP15* as a key regulator of follicular growth and oocyte competence [47]. Complementary scRNA-seq of bovine embryos mapped lineage-specific transcriptional programs driving early embryogenesis and implantation potential [50].

Within this framework, functional validation should prioritize three actionable levels—upstream axis regulators, ovarian–follicular checkpoints, and stage-specific regulators of spermatogenesis—using a stringent inclusion rule of cross-layer replication together with colocalization or mediation support, cell type-resolved localization, and positive functional perturbation. This disciplined gatekeeping curbs false positives inherent to list-like reporting and focuses resources on loci with mechanistic credibility and realistic potential to improve fertility EBVs and selection indices in beef cattle.

### 2.4. Discovery-Stage Signals for Environmental Adaptation and Resilience

Environmental adaptation and resilience in cattle address heritable responses to long-term ecological pressures—heat and cold stress, aridity/humidity, pathogens, and grazing intensity. The phenotypic landscape spans thermoregulation and heat dissipation, energy metabolism and adipose remodeling, skin/coat and the cornified layer, membrane stability and ion homeostasis, and systems-level reprogramming of immune and oxidative-stress pathways [58,59]. Genetically, polygenicity coexists with structural variation: beyond conventional SNP associations, copy-number variation (CNV) and long-range regulation recur in tropical and arid-climate adaptation [60,61]. Methodologically, mainstream practice increasingly couples selection scans and structural-variant discovery with tissue- and environment-specific expression, metabolomic profiling, and genotype-by-environment interactions (G × E) modeling, extending toward region-specific genomic prediction—a pipeline that grounds the climate–genotype–phenotype linkage in testable candidates and deployable breeding decisions [62,63]. In this review, resilience is considered an integrative health-related complex trait, encompassing resistance and tolerance to environmental, metabolic, and infectious challenges that jointly determine animal robustness [64,65].

Evidence typically aggregates along two complementary tracks. A cold-adaptation track centers on thermogenesis, progresses through energy metabolism, and culminates in adipose browning, where concordant population and transcriptomic signals highlight genes such as *PRDM16* and *AQP3*/*AQP7* [66,67,68,69,70]. A heat-adaptation track emphasizes membrane stability, links with ion homeostasis, and converges on heat-stress responses, with repeated support for *MYO1A* and *TECPR2* in heat-tolerance studies [66,67,68]. Structural layers frequently reconcile cross-population reproducibility: in indicine lineages, multi-copy expansions at the *FADS2* family—implicated in fatty-acid desaturation—point to a family-level regulatory mechanism in ecological adaptation [60]. Integrative single-cell eQTL analyses further pinpointed regulatory variants underlying cellular stress-response networks and adaptive thermoregulation [9].

A practical workflow is articulated in four steps. First, nominate candidates via selection scans and structural-variant mapping. Second, integrate environmental association analyses using covariates such as the Temperature–Humidity Index (THI), altitude/partial pressure of oxygen, and aridity/humidity indices; combine this with tissue- and environment-specific expression to pinpoint loci sensitive to temperature or hypoxia. Third, add metabolomics—fatty-acid composition and antioxidant metabolites—as mediators to position pathways (e.g., desaturation and redox buffering) between genotype and phenotype. Finally, validate direction and magnitude with multi-site, multi-season reaction norms [62,71] and translate the outcomes into regionalized selection and breed–environment matching schemes. This end-to-end design elevates dispersed signals into actionable evidence for resilience and supports genetic gains that remain robust across climates and management systems.

While single-omics analyses can identify statistically significant loci and pathways, many signals remain context-dependent and subject to confounding by linkage disequilibrium, environmental covariates, and regulatory pleiotropy. Therefore, cross-omics integration and causal inference are essential to disentangle these effects, strengthen mechanistic interpretation, and enhance the translatability of candidate genes and pathways into breeding applications.

## 3. Cross-Omics Integration and Causal Localization Move Candidates Toward Translation

Instead of listing individual signals, we adopt a tiered approach to strengthen evidence step by step. In this review, causal localization denotes the process of refining statistical associations to mechanistically interpretable loci by integrating regulatory colocalization, mediation, and network centrality evidence, followed by experimental or phenotypic validation. First, we identify statistical associations. Next, we assess colocalization and regulatory support. We then incorporate network and causal analyses to reduce false positives. Finally, validated candidates are translated into practical breeding applications. In practice, containerized and version-controlled workflows—such as Snakemake or Nextflow—ensure end-to-end reproducibility from raw data to figures. We set non-negotiable requirements for batch-effect correction, minimum-information records for samples and environments, cross-omics identifier mapping, and auditable quality control, so that candidates replicated across populations can be incorporated into selection indices [72,73]. For ease of use, concise and reproducible checklists aligned with the four trait classes are provided in Table 1, Table 2, Table 3 and Table 4.

In the sections that follow, we align growth, meat quality, reproduction, and environmental adaptation with this evidence pathway and specify the interface to application endpoints for each trait class.

### 3.1. Integrated Multi-Omics Evidence for Growth and Development

Growth traits (body size, body weight, and carcass indicators) typically reflect cooperating, polygenic clusters, calling for a layered strategy that progresses from cross-population association, passes through regulatory colocalization and network/causality, and culminates in functional validation. First, conduct GWAS or meta-GWAS in diverse ancestries to leverage lineage differences and attenuate linkage disequilibrium (LD) tails, exemplified by *PLAG1*–*LCORL*/*NCAPG* [22,30]. Second, perform eQTL/TWAS and splicing localization in muscle/bone, integrating ATAC-seq and DNA methylation to delineate cis-elements and avoid bystander misassignment [12,16,54]. Third, combine WGCNA/gene regulatory network (GRN) with colocalization/mediation to trace paths from variant, through expression, to phenotype and to rank module centrality/redundancy in myogenesis, ECM remodeling, and skeletal development [31]. Along this main line, assign informative priors to admitted loci within single-step genomic best linear unbiased prediction (ssGBLUP) or Bayesian regression with categorical prior (BayesRC) and link them to growth–carcass indices.

Critically, genes such as *IMPAD1*, *PENK*, *STC2*, and *CPEB4* have been resolved from multi-gene signals inflated by LD or bystanders and, together with *CSMD3*, *LAP3*, *SYN3*, *FAM19A5*, and *TIMP3*, collectively account for the genetic basis of body-size and bone-growth variation [32,33,34].

For prioritization and validation/application, we recommend an inclusion threshold of cross-population replication, colocalization or mediation support, network centrality, and concordant functional readouts. Validation should mirror the target traits and span both in vitro and in vivo settings: use CRISPRi/a or mini-gene splicing perturbations in myogenic/chondrogenic differentiation systems with readouts aligned to fiber-type composition, ECM markers, and osteogenic signals; at the animal level, integrate slaughter or ultrasound records and assign informative priors to admitted loci within ssGBLUP or BayesRC, feeding into growth–carcass selection indices [68,74,75]. As a capstone linking network or causality to functional validation, fine-mapping that uses ancestry-recombination maps together with sweep signals has elevated *LCORL* and *STC2* to likely causal variants; gene-edited mouse models report large effects on body weight (~11%), compressing broad associations into verifiable and translatable candidates [76]. To enhance clarity in translating multi-omics evidence into practical applications, we summarize in Table 1 how growth-related modules—spanning association, regulation, and network integration—connect to breeding evaluation, in-farm phenotyping, and workflow reproducibility. Each evidence block is linked to its operational endpoint, forming a minimal and reproducible checklist for index integration and standardized recording. Table 1 aligns four evidence blocks—body-size/bone-growth hubs, ECM and fiber formation, cooperating gene clusters, and methods/workflow—with application anchors to provide a minimal, reproducible checklist for index integration and field recording. The genes shown in Table 1, Table 2, Table 3 and Table 4 are therefore illustrative exemplars, representing the most reproducible and biologically interpretable signals within each module rather than exhaustive lists of all significant loci.

**Table 1:** Growth and development—evidence chain and application anchors.

Module or Pathway	Representative Candidates	Evidence	Suggested Application	Practical Application (Breeding/Monitoring/Management)	References
Body-size and bone-growth hub	*PLAG1*–*LCORL*/*NCAPG*	Cross-ancestry GWAS and meta-GWAS followed by TWAS and colocalization (PP4 increased); tissue expression consistent	Stability anchor to benchmark other loci; track effect direction	Breeding evaluation: benchmark loci for growth index; Farm monitoring: track growth curve consistency	[22,30]
ECM and muscle-fiber formation	*IMPAD1*; *PENK*; *STC2*; *CPEB4*	WGCNA or GRN centrality increased; mediation; colocalization with body-size traits	Add informative priors in indices for size and bone mass	Breeding: informative priors in ssGBLUP; Monitoring: bone-density/ECM markers	[31,32,33,34]
Cooperating gene clusters	*CSMD3*; *LAP3*; *SYN3*; *FAM19A5*; *TIMP3*	Module explains variation with tissue eQTL support	Modular weighting for body-size and bone profile	Management: integrate cluster weighting into carcass evaluation pipeline	[32,33,34]
Methods and workflow	eQTL; TWAS; ATAC; methylation	From association, through regulation and network constraints, to validation; containerized; batch control, QC, and ID mapping	Reproducible pipeline; Minimum Information sheets	Batch management: ensure reproducible QC, ID mapping, and metadata tracking	[5,7,9]

### 3.2. Integrated Multi-Omics Evidence for Carcass and Meat Quality

During finishing, the core objective is to increase IMF content and to optimize its deposition pattern, thereby improving tenderness, flavor, and grading premiums. Across data layers, the most consistently replicated functional backbone runs from lipid-droplet formation through fatty-acid transport and desaturation. Because fiber type is closely tied to meat quality, joint transcriptomic–proteomic–metabolomic analyses have repeatedly revealed cross-layer concordant pathway signals and reinforced the explanatory power of integrative omics for beef-quality traits [77]. Operationally, we recommend first conducting joint enrichment in longissimus dorsi and adipose tissue using transcriptome, methylome, and metabolome data to identify a three-segment network comprising metabolic enzymes, transporters, and regulators (the canonical *FABP4*/*SCD*/*ADIRF* axis). Subsequently, apply GWAS/TWAS colocalization to collapse module-level signals onto experimentally tractable genes or variants [35,36,37,38,77,78].

In parallel, anchor candidate genes to field-accessible phenotypes—IMF and fatty-acid profile to operationalize lipid-handling biology [40], and instrument-based readouts such as Warner–Bratzler shear force and near-infrared (NIR) spectroscopy to quantify tenderness in practice [41]. This creates a monitorable and verifiable feedback loop between molecular variation and measurable traits. Prior findings across breeds corroborate associations of *XKR4* with subcutaneous backfat thickness [44], while *PLIN1*/*SLCO4C1* and the *SLC16A7*/*SLC22* families track with marbling and lipid-metabolism phenotypes; coordinated methylation–transcript coupling further implicates *GNAS*, *PDE4B*, *EPCAM*, and *EBF3* in tenderness-related pathways [79,80]. Collectively, these associations are consistent with recent multi-omics findings, which have clarified the lipid-metabolic regulation underlying meat quality by revealing coordinated control of fatty-acid desaturation and lipid-droplet remodeling in muscle fibers [77].

Prioritization follows a joint criterion that combines network centrality with contribution to measurable phenotypes. For validation, treat the fatty-acid profile and the desaturation index as mediating phenotypes and apply causal mediation analysis to test the placement of *SCD*/*FABP4* and related nodes within the pathway [80,81]. Advancement to functional and translational stages should require candidates to be monitorable during finishing, to deliver marginal gains for index weighting, and to remain robust across cohorts and diet regimens; genetic evaluations should explicitly record G × E to enable stratified weights for regional finishing programs [40,41,44]. To avoid mistaking nutrition or age differences for genetic effects, standardize a Finishing Minimum Information Sheet at the workflow level and model batch as a random effect with a diet × genotype interaction term [72,73]. Table 2 aligns lipid-droplet biogenesis, transport, desaturation, fat distribution/marbling, subcutaneous fat thickness, and implementation/evaluation into an evidence-to-application map that is directly linked to mediating phenotypes and minimum-information requirements.

**Table 2:** Carcass and meat quality—lipid droplet, transport, and desaturation axis.

Module or Pathway	Representative Candidates	Evidence	Suggested Application	Practical Application (Breeding/Monitoring/Management)	References
Lipid-droplet biogenesis, transport, and desaturation (three-segment)	*FABP4*; *SCD*; *ADIRF*	Joint enrichment in transcriptome, methylome, and metabolome; concordant with IMF percentage and fatty-acid profile	Use fatty-acid profile and desaturation index as mediating phenotypes for validation	Breeding: include desaturation index as genomic weight for meat-quality selection; Monitoring: track fatty-acid profile and IMF % via NIR or biochemical assays; Management: adjust feeding regime based on IMF trend	[35,36,37,38,78]
Fat distribution and marbling	*PLIN1*; *SLCO4C1*; *SLC16A7*; *SLC22* family	Coordinated epigenetic and transcript coupling; covaries with marbling and tenderness	Field Warner–Bratzler shear force and near-infrared monitoring; link to indices	Breeding: integrate marbling score and tenderness into multi-trait selection; Monitoring: on-farm infrared sensors for carcass grading; Management: feedback loop between carcass data and finishing diets	[79]
Subcutaneous backfat thickness	*XKR4*	Multi-breed association replicated	Add distribution weight in the index	Breeding: use as stability marker for fat deposition; Monitoring: ultrasound or digital imaging for back-fat tracking; Management: optimize energy balance in finishing phase	[44]
Implementation and evaluation	IMF percentage; MUFA to SFA ratio; C18:1; WBSF; NIR	Cross-layer colocalization leading to a monitorable-phenotype loop; G × E recorded	Finishing Minimum Information Sheet; model batch as a random effect with a diet by genotype interaction	Breeding: validate across herds to refine index weighting; Monitoring: collect standardized finishing data; Management: apply batch QC and diet-genotype recording via unified templates	[40,41,72,73]

### 3.3. Integrated Multi-Omics Evidence for Reproductive Traits

Because reproductive traits (APU, AFC, HCR/HP, CCR, CI, stayability, and male SC and semen quality) are low-heritability and highly sensitive to environment and nutrition, GWAS/TWAS hits are best mapped onto a coordinate system defined by cell type and developmental stage derived from single-cell or spatial transcriptomics, then fused with epigenomic layers (ATAC-seq, DNA methylation, m6A, and noncoding RNA) to pinpoint actionable regulatory nodes [9,23,47,50]. Along the axis-level neuroendocrine control, transcriptomic and co-expression evidence repeatedly implicates upstream nodes such as *POMC*, *CHGA*, and *PENK* in the initiation of puberty [46]. Within the ovarian/uterine microenvironment, coupling among m6A, alternative splicing, and noncoding RNAs orchestrates folliculogenesis and implantation; in cattle, an *ALKBH5-BMP15* m6A mechanism has been associated with puberty timing [47]. On the male side, stage specificity during spermatogenesis is shaped by circRNA-mediated buffering together with chromatin accessibility [57,81].

Candidate prioritization should proceed in layers—from upstream axis regulators, through key follicular checkpoints, and on to stage-specific regulators of spermatogenesis—and validation should combine in situ perturbation and rescue with cross-platform models: interventions in granulosa cells, cumulus–oocyte complexes (COCs), and uterine epithelium on the female side, and organoids or in vitro spermatogenesis systems on the male side. For application, align molecular phenotypes (e.g., puberty program score, follicle maturation index, spermatogenesis homeostasis index) with production metrics such as conception rates, open days, and CI, and incorporate them into reproductive indices [46]. Statistically, tighten covariate control for season, nutrition, and health status, and use multi-scenario replication to avoid mistaking management differences for genetic effects [23]. For implementation, Table 3 links four evidence layers—neuroendocrine upstream, ovarian/uterine microenvironment, male gametogenesis, and recording/modeling—to index endpoints (APU/AFC/CI/stayability).

**Table 3:** Reproductive traits—multilayer integration to indices.

Module or Layer	Representative Candidates	Evidence	Suggested Application	Practical Application (Breeding/Monitoring/Management)	References
Neuroendocrine upstream	*POMC*; *CHGA*; *PENK*	Transcriptome with co-expression implicates puberty initiation; TWAS and colocalization support	Build a Puberty Program Score mapped to APU and AFC	Breeding: include puberty score as genomic predictor; Monitoring: measure puberty onset or cyclicity via hormonal assays; Management: schedule synchronization protocols by maturity stage	[46]
Ovarian and uterine microenvironment	*ALKBH5-BMP15* (m6A)	m6A with splicing and noncoding RNA coupling; associated with puberty timing	Perturbation and rescue in cumulus–oocyte complexes and granulosa cells	Breeding: prioritize fertility alleles in index; Monitoring: track follicle growth, oocyte quality; Management: nutritional and hormonal adjustment to support maturation	[9,47]
Male gametogenesis	circRNA–target networks	Single-cell RNA sequencing and single-cell ATAC or CUT and Tag show stage-specific regulation	Validate with organoids and in vitro spermatogenesis systems	Breeding: select bulls based on spermatogenic stability markers; Monitoring: semen-quality scoring (motility, circRNA biomarkers); Management: manage temperature and stress conditions in AI centers	[57,81]
Recording and modeling	APU; AFC; HCR or HP; CCR; CI; SC	Low heritability (h^2^) requires large cohorts and standardization; control season, nutrition, and health status	Include stayability and open days in indices	Breeding: integrate stayability in lifetime-productivity index; Monitoring: record open days and conception rate; Management: implement reproductive-data logging and seasonal adjustment	[23]

### 3.4. Integrated Multi-Omics Evidence for Environmental Adaptation and Resilience

Evidence for environmental adaptation can be integrated along two principal tracks: a cold-adaptation track that runs from thermogenesis, through energy metabolism, and on to adipose browning, and a heat-adaptation track that begins with membrane stability, proceeds through ion homeostasis, and culminates in heat-stress responses. In cold-adapted populations, *PRDM16* and *AQP3*/*AQP7* are repeatedly enriched; in heat-tolerance studies, *MYO1A* and *TECPR2* are recurrently implicated [66,68,69,70]. Genetically, adaptation is typically co-driven by polygenic architectures and structural variants (SVs, including CNVs). In East Asian taurine cattle, hypoxia-response loci such as *EPAS1* and *EGLN1* operate against a background of introgression-associated structural variation and long-range regulation layered on gradients of altitude and the partial pressure of oxygen, jointly shaping regional signals from humid–monsoonal to high-cold ecologies. This pattern is consistent with a model in which structural variation supports broad adaptive mechanisms rather than lineage-specific peculiarities [3,43,82]. Accordingly, discovery should begin with selection scans and structural-variant mapping, followed by environmental association analyses (e.g., THI, altitude/partial pressure of oxygen, aridity–humidity indices) and tissue- or environment-specific expression to identify temperature- or hypoxia-sensitive genes; metabolomics can then provide mediating evidence via fatty-acid composition and energy-substrate preferences, with final confirmation through cross-ecology consistency tests [62,63].

Prioritization should combine multi-site and multi-season reaction norms (slope and curvature), network centrality, and monitorable phenotypes. Loci that replicate across contexts and occupy central positions in metabolic or stress-response pathways should advance first into regionalized selection and breed–environment matching. In practice, a layered genotyping panel (a universal core plus ecozone-specific subpanels) can be paired with informative priors in GBLUP/ssGBLUP [13]. To avoid confounding ancestry with adaptation, sampling designs should incorporate ecological stratification and explicit ancestry correction, and wherever possible, pangenome/graph references should be used to reduce reference bias [3,72]. Table 4 consolidates the cold and heat tracks, structural variation, and regional translation into an executable roadmap for deployment and diet stratification.

**Table 4:** Environmental adaptation and resilience—eco-adaptation framework.

Module or Pathway	Representative Candidates	Evidence	Suggested Application	Practical Application (Breeding/Monitoring/Management)	References
Cold-adaptation track	*PRDM16*; *AQP3*; *AQP7*	Population signals consistent with cold-tolerance phenotypes; single-cell and epigenomic support	Metabolomics informing browning and energy-substrate preferences	Breeding: include thermogenic and lipid-oxidation markers in adaptive index; Monitoring: measure body-temperature resilience and metabolite profile in cold season; Management: optimize feeding and housing for cold regions	[66,75,83]
Heat-adaptation track	*MYO1A*; *TECPR2*	Repeated across heat-tolerance studies; aligns with THI, body temperature, and behavior	Joint modeling with THI and behavioral phenotypes	Breeding: incorporate heat-tolerance loci in tropical index; Monitoring: track THI, panting score, body temp; Management: implement shade/cooling/watering schedule by genotype	[68,69,70]
Structural variation	*EPAS1*; *EGLN1*	Introgression with structural variation and distal regulation consistent with altitude and the partial pressure of oxygen; tissue and environment-specific expression	Selection scans, environmental association, and regulatory evidence; multi-site and multi-season reaction norms	Breeding: select for hypoxia-resistant genotypes; Monitoring: use hematologic and oxygen-saturation indicators; Management: plan herd movement or breeding by altitude	[3,43,82]
Regional translation	THI, altitude, and aridity–humidity by genotype	Environment-specific expression with metabolomics, G × E prediction, and reaction-norm validation	Design regional deployment and diet stratification schemes	Breeding: establish ecozone-specific sub-panels; Monitoring: link genotype with local THI records; Management: tailor diet and breeding schedule per region	[62,63]

## 4. Multi-Omics Evidence Chains, G × E, and Functional Validation Connect Association to Causality and Translation for Breeding Applications

On the application side, dispersed omics signals should be organized into a traceable evidence chain supported by a minimal and reproducible workflow. We first capture stable associations through cross-population and multi-environment genome-wide association studies. We then perform regulatory colocalization and expression-mediated tests using expression quantitative trait loci, splicing quantitative trait loci, and transcriptome-wide association studies, and converge on cis-regulatory elements with epigenomic evidence such as open-chromatin accessibility and DNA methylation. Next, we add network analyses and causal inference—co-expression and regulatory networks, causal graphs, and mediation analysis—to assess directionality and coherence, followed by functional readouts at both cellular and animal levels. Finally, we align monitorable mediating phenotypes—for example, the fatty-acid profile or desaturation index, a puberty-program score, and thermogenesis or stress indicators—with genetic evaluation and selection indices. This stepwise framework provides explicit entry and triage criteria and auditable quality-control checkpoints that curb false positives and improve robustness across contexts [3,5,7,9,17,18].

### 4.1. A Framework for Causal Inference and Localization Brings Correlation Closer to Causation

We advocate a layered evidence chain to prioritize putative causal loci. First, replicate statistical associations across divergent ancestries and independent populations, while conducting LD refinement and Bayesian posterior–factor or credible-set evaluation in parallel. Second, at the same locus, integrate eQTL, sQTL, and chromatin QTL (cQTL) with trait associations, and apply regulatory co-mapping together with expression-mediated tests (for example, TWAS) to strengthen functional credibility [13,19,21]. At the reference-genome level, employ pangenome or graph references to make SVs (including CNVs and insertions) explicit, thereby reducing reference bias and missed variation; population studies in *East Asian* cattle demonstrate a systematic correspondence between structural variation, introgression events, and local environmental adaptation [3]. CNV, as a structural variant affecting gene dosage and regulatory architecture, is incorporated into the causal-inference chain via colocalization and mediation analyses that trace dosage-dependent transcriptional effects to phenotypic outcomes [84,85]. Accordingly, we propose an evidence-grade ladder with four rungs: statistical association; multi-omics colocalization with cross-ancestry concordance; functional-annotation support (including epigenomic and regulatory context); and cellular or animal validation. Only loci supported at multiple rungs should enter the causal-priority shortlist.

### 4.2. Multiscale Functional Validation Turns Statistical Signals into Biological Mechanisms

Interpreting variants within their cell-type-specific and developmental-stage-resolved context is essential for building a continuous chain from variant, through regulatory effects at the cell or tissue level, and onward to phenotype. Joint analyses that couple single-cell transcriptomics with chromatin-accessibility profiling can resolve lineage trajectories and regulatory modules for satellite cells and fibro-adipogenic progenitors [75,83]. To pinpoint causal variants within specific cell types, single-cell transcriptomic and chromatin-accessibility data can be jointly analyzed to identify expression or co-accessibility quantitative trait loci (sc-eQTLs/c-QTLs) confined to defined cell clusters. Loci showing strong colocalization with population-level GWAS or TWAS signals are prioritized as candidate causal mutations [11,51,53,83]. Validation is then performed in the corresponding lineage or organoid system using targeted perturbations—such as CRISPR interference/activation or enhancer editing—to test transcriptional and phenotypic consequences [17,51,52]. These single-cell-resolved experiments confirm directionality and specificity, providing a mechanistic bridge from variant to cell-type function and ultimately to phenotypic manifestation [51,83].

On this foundation, integrating Ribo-seq, proteomics, and metabolomics extends transcriptional associations into protein interaction and metabolic networks, thereby closing the evidence loop across molecular, cellular, and tissue scales. For adaptive traits, as one illustration, *PRDM16* shows concordant population-level signals with cold-tolerance phenotypes [66], and structural variants may act as switch-like regulators in multi-environment adaptation [3]. Taken together, these principles motivate downstream targeted editing (CRISPR/Cas or CRISPRi/a), enhancer and 3′UTR functional assays, and Perturb-seq, which accelerate the conversion of statistical hits into verified mechanisms and, ultimately, translatable targets.

### 4.3. G × E and Reaction Norms Bring the Environment into the Causal Chain

Complex traits in livestock are often substantially shaped by G × E. We recommend building standardized environmental metadata from quantifiable indicators—such as the temperature–humidity index (THI), altitude and the partial pressure of oxygen, diet formulations, and pathogen exposure—and aligning these records with production outcomes. For estimation, use random-regression and multi-trait-by-environment joint models, and consider sparse or hierarchical priors to accommodate high-dimensional environmental factors. Systematic reviews report repeated evidence of genotype–climate interactions for production traits and for subsets of health and reproductive traits, although the latter two categories still require larger samples and broader spatiotemporal coverage [86,87,88]. For field validation, we advise multi-site and multi-season replication to fit reaction-norm curves (slope and curvature) and to link these parameters to economic weights, thereby identifying environment-specific optimal genotypes. In this context, “bringing the environment into the causal chain” denotes incorporating environmental factors as explicit mediators or modifiers linking genotype, molecular traits, and phenotype. Reaction norms statistically quantify how environmental gradients modulate genetic effects, thereby translating biological G × E mechanisms into predictive models of performance across environments [86,87,88].

To explicitly model G × E at the marker or omics-feature level, we adopt a reaction-norm random-regression framework in which marker effects vary as functions of environmental covariates. The model can be written as

y_i_ = μ + Σ_j_ (x_ij_ β_j0_ + x_ij_ β_j1_ E_i_ + x_ij_ β_j2_ E_i_^2^ + …) + Σ_k_ z_ik_ γ_k_ + ε_i_.

Here, β_j0_ represents the baseline additive effect, whereas β_j1_ and β_j2_ measure linear and nonlinear sensitivities to environmental gradients (e.g., THI, altitude, nutritional plane). These parameters correspond to reaction-norm slope and curvature, which quantify genotype-specific environmental plasticity and are widely used in livestock G × E modeling [89].

Biologically informed priors can be constructed by assigning larger prior variances or higher prior inclusion probabilities to variants supported by multi-omics evidence such as eQTL or regulatory annotation, as implemented in the BayesRC framework [90]. In ssGBLUP, equivalent biological weighting is achieved by modifying the genomic relationship matrix to up-weight validated loci [91].This strategy strengthens predictive accuracy while maintaining statistical rigor across heterogeneous environments.

### 4.4. Multi-Omics Mechanisms Are Translated into Breeding Decisions

Once certain loci reach a near-causal or high-confidence threshold along the evidence chain, group these loci—together with key SVs and functional SNPs—into a layered genotyping panel composed of a universal core and ecozone-specific subpanels tailored to distinct production ecologies [13]. Validated loci supported by multi-omics evidence can be incorporated into breeding programs through weighted genomic models and indicator traits. In single-step GBLUP or Bayesian frameworks, biologically confirmed variants receive informative priors that reflect their functional relevance and stability across environments [92,93]. Multi-omics-derived indicators, such as fatty-acid composition or thermogenic capacity, can improve prediction accuracy for complex traits [94]. In practice, customized genomic panels combine a universal SNP core with trait- or region-specific subpanels for resilience and product quality [58,62]. Successful examples include the integration of *PLAG1*–*LCORL* and *SCD*–*FABP4* variants for carcass and fat-quality selection and *EPAS1*–*EGLN1* variants for high-altitude adaptation [25,76]. These approaches demonstrate that multi-omics findings can be effectively transformed into actionable components of genomic selection indices.

Within GBLUP, ssGBLUP, or BayesRC, assign informative priors to these loci and incorporate multi-omics-derived intermediate phenotypes—such as a brown-fat thermogenesis score, a skin-barrier lipid-metabolism index, and an immune-response index—as intermediate (molecular) phenotypes to improve predictive robustness and cross-environment generalization. The resulting product is a multi-omics candidate-parent scorecard paired with regional mate-allocation schemes, supported by economic-weight evaluations against key performance indicators (e.g., ADG, FCR, fertility rate, and heat- or cold-stress loss rates). Field indicators are then compared with model predictions to complete a closed-loop test and to enable rolling calibration of the selection index.

In parallel, machine learning and deep learning approaches are increasingly being adopted to model nonlinear, high-dimensional, and cross-layer relationships in multi-omics data. Algorithms such as random forests, gradient boosting, and Bayesian ensemble models can capture nonlinear genotype–phenotype interactions and multi-locus effects that are often missed by conventional linear models [95,96]. Deep learning architectures—including convolutional neural networks (CNNs), recurrent neural networks (RNNs), and graph neural networks (GNNs)—extract hierarchical regulatory features and spatial or temporal dependencies among omics layers, linking chromatin accessibility, transcriptional regulation, and phenotypic variation [97]. In livestock breeding, these models have improved the prediction of genomic estimated breeding values (GEBVs) by integrating genomic, transcriptomic, and environmental covariates, while autoencoder- and attention-based models can identify latent representations that enhance robustness across populations [98]. Combining deep learning with causal inference and reaction-norm modeling provides a scalable route for translating multi-omics information into more accurate and environment-aware genetic evaluations [99].

The refined causal-inference framework provides a direct route from variant discovery to breeding application. By integrating SNPs, indels, and CNVs with regulatory and expression evidence, it identifies validated loci that can be incorporated into genomic prediction and selection indices. This linkage ensures that multi-omics findings are not only statistically sound but also operationally useful for breeding evaluation and decision-making.

## 5. Conclusions 

**Figure 1:**
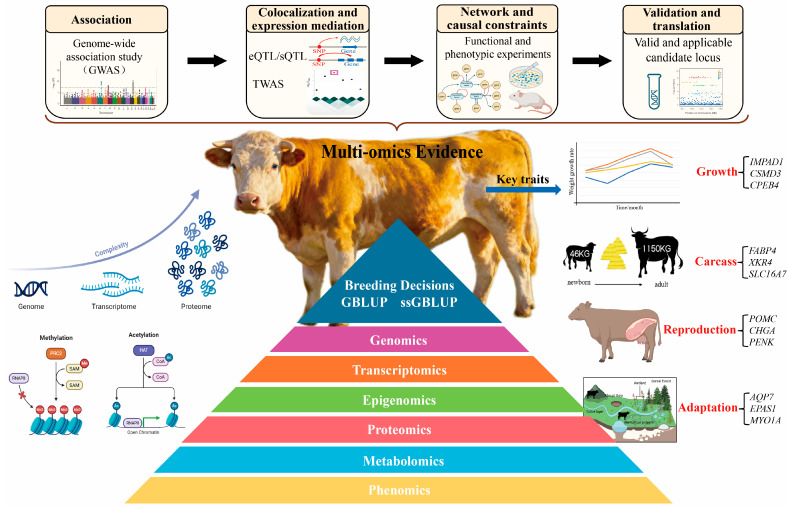
Conceptual framework illustrating the progressive integration of multi-omics evidence—from association to colocalization, causation, and transformation. Each analytical stage is associated with specific evidence levels and entry thresholds: genome-wide association for variant-level association; spatial or regulatory concordance for co-localization; mediation or perturbation-based validation for causal inference; and breeding utility for transformation.

Focusing on complex traits in beef cattle, we outline a practical pathway from multi-omics evidence chains to selection-index integration: cross-population and multi-environment associations guide discovery; colocalization and regulatory evidence narrow and prioritize candidates; network- and causality-based frameworks integrate multilayer information; and functional together with phenotypic readouts complete interpretability, thereby turning stable, reproducible markers and genes into actionable breeding information. Structurally, we organize the narrative around two application lines—growth and efficiency, and carcass and meat quality—to demonstrate a coherent progression from signals, through mechanisms, to applications. Figure 1 consolidates this pathway—aligning evidence tiers, as well as mediating phenotypes, G × E, and index integration—so that practitioners can retrace each link from signals to decisions.

For deployment, we recommend piloting with a focused set of high-confidence candidates, paired with enhanced production recording and calibrated economic weights, and supported by containerized, cross-platform reproducible workflows and auditable QC norms, thereby shortening the path from evidentiary synthesis to production rollout. Looking ahead, priorities include raising the spatiotemporal resolution and verifiability of the evidence chain, completing an engineered pipeline from data governance to model deployment, and adopting dynamic weighting and adaptive strategies under explicit risk–benefit trade-offs to ensure robust genetic gain across ecologies and management systems. Taken together, this roadmap provides an operational framework—and concrete levers—for translating dispersed multi-omics information into clear, executable, and scalable breeding decisions.

## Data Availability

No new data were created or analyzed in this study. Data sharing is not applicable to this article.

## References

[1] Daniel P., Natalia S., Nicolazzi E.L., Machugh D.E., Park S.D.E., Licia C., Rodrigo M., Bruford M.W., Pablo O.T. (2018). Domestication of cattle: Two or three events?. Evol. Appl..

[2] Decker J.E., McKay S.D., Rolf M.M., Kim J., Molina Alcala A., Sonstegard T.S., Hanotte O., Gotherstrom A., Seabury C.M., Praharani L. (2014). Worldwide patterns of ancestry, divergence, and admixture in domesticated cattle. PLoS Genet..

[3] Xia X., Zhang F., Li S., Luo X., Peng L., Dong Z., Pausch H., Leonard A.S., Crysnanto D., Wang S. (2023). Structural variation and introgression from wild populations in East Asian cattle genomes confer adaptation to local environment. Genome Biol..

[4] FAO (2024). Meat Market Review: Overview of Global Market Developments in 2023.

[5] Hasin Y., Seldin M., Lusis A. (2017). Multi-omics approaches to disease. Genome Biol..

[6] Stefan G., Kevin C., Washam C.L., Allen G., Jordan B., Robeson M.S., Byrum S.D. (2021). Multi-omics data integration considerations and study design for biological systems and disease. Mol. Omics.

[7] Picard M., Scott-Boyer M.P., Bodein A., Olivier P., Droit A. (2021). Integration strategies of multi-omics data for machine learning analysis. Comput. Struct. Biotechnol. J..

[8] Oakley A.E., Clifton D.K., Steiner R.A. (2009). Kisspeptin signaling in the brain. Endocr. Rev..

[9] Wang L., Khunsriraksakul C., Markus H., Chen D., Zhang F., Chen F., Zhan X., Carrel L., Liu D.J., Jiang B. (2024). Integrating single cell expression quantitative trait loci summary statistics to understand complex trait risk genes. Nat. Commun..

[10] Fontanesi L. (2016). Metabolomics and livestock genomics: Insights into a phenotyping frontier and its applications in animal breeding. Anim. Front..

[11] Wadood A.A., Bordbar F., Zhang X. (2025). Integrating omics approaches in livestock biotechnology: Innovations in production and reproductive efficiency. Front. Anim. Sci..

[12] Cai W., Zhang Y., Chang T., Wang Z., Zhu B., Chen Y., Gao X., Xu L., Zhang L., Gao H. (2023). The eQTL colocalization and transcriptome-wide association study identify potentially causal genes responsible for economic traits in Simmental beef cattle. J. Anim. Sci. Biotechnol..

[13] Gao Z., Lu Y., Chong Y., Li M., Hong J., Wu J., Wu D., Xi D., Deng W. (2024). Beef cattle genome project: Advances in genome sequencing, assembly, and functional genes discovery. Int. J. Mol. Sci..

[14] Jitjumnong J., Taweechaipaisankul A., Lin J.C., Wongchanla S., Chuwatthanakhajorn S., Lin C.J., Khang L.T.P., Linh N.V., Sangsawad P., Dinh-Hung N. (2025). An overview of advancements in proteomic approaches to enhance livestock production and aquaculture. Animals.

[15] Langfelder P., Horvath S. (2008). WGCNA: An R package for weighted correlation network analysis. BMC Bioinform..

[16] Smith J.L., Wilson M.L., Nilson S.M., Rowan T.N., Schnabel R.D., Decker J.E., Seabury C.M. (2022). Genome-wide association and genotype by environment interactions for growth traits in U.S. Red Angus cattle. BMC Genom..

[17] Fulco C.P., Munschauer M., Anyoha R., Munson G., Grossman S.R., Perez E.M., Kane M., Cleary B., Lander E.S., Engreitz J.M. (2016). Systematic mapping of functional enhancer–promoter connections with CRISPR interference. Science.

[18] Zaccara S., Ries R.J., Jaffrey S.R. (2019). Reading, writing and erasing mRNA methylation. Nat. Rev. Mol. Cell Biol..

[19] Giambartolomei C., Vukcevic D., E Schadt E., Franke L., Hingorani A.D., Wallace C., Plagnol V. (2014). Bayesian test for colocalisation between pairs of genetic association studies using summary statistics. PLoS Genet..

[20] Barbeira A.N., Pividori M., Zheng J., E Wheeler H., Nicolae D.L., Im H.K. (2019). Integrating predicted transcriptome from multiple tissues improves association detection. PLoS Genet..

[21] Gusev A., Ko A., Shi H., Bhatia G., Chung W., Penninx B.W.J.H., Jansen R., De Geus E.J.C., Boomsma D.I., Wright F.A. (2016). Integrative approaches for large-scale transcriptome-wide association studies. Nat. Genet..

[22] Takasuga A. (2015). PLAG1 and NCAPG-LCORL in livestock. Anim. Sci. J..

[23] Kenny D.A., Fitzsimons C., Waters S.M., McGee M. (2018). Invited review: Improving feed efficiency of beef cattle—The current state of the art and future challenges. Animal.

[24] Berry D.P., Crowley J.J. (2012). Residual intake and body weight gain: A new measure of efficiency in growing cattle. J. Anim. Sci..

[25] Berry D.P., Crowley J.J. (2013). Cell biology symposium: Genetics of feed efficiency in dairy and beef cattle. J. Anim. Sci..

[26] Mulhall S.A., Sleator R.D., Evans R.D., Berry D.P., Twomey A.J. (2024). Effect on prime animal beef merit from breeding solely for lighter dairy cows. J. Dairy Sci..

[27] Basiel B.L., Felix T.L. (2022). Board Invited Review: Crossbreeding beef × dairy cattle for the modern beef production system. Transl. Anim. Sci..

[28] Weik F., Hickson R.E., Morris S.T., Garrick D.J., Archer J.A. (2021). Genetic parameters for growth, ultrasound and carcass traits in New Zealand beef cattle and their correlations with maternal performance. Animals.

[29] Do C., Park B., Kim S., Choi T., Yang B., Park S., HyungJun S. (2016). Genetic parameter estimates of carcass traits under national scale breeding scheme for beef cattle. Asian Australas. J. Anim. Sci..

[30] Majeres L.E., Dilger A.C., Shike D.W., McCann J.C., Beever J.E. (2024). Defining a haplotype encompassing the LCORL-NCAPG locus associated with increased lean growth in beef cattle. Genes.

[31] An B., Xia J., Chang T., Wang X., Xu L., Zhang L., Gao X., Chen Y., Li J., Gao H. (2019). Genome-wide association study reveals candidate genes associated with body measurement traits in Chinese wagyu beef cattle. Anim. Genet..

[32] Wu D.-D., Yang C.-P., Wang M.-S., Dong K.-Z., Yan D.-W., Hao Z.-Q., Fan S.-Q., Chu S.-Z., Shen Q.-S., Jiang L.-P. (2020). Convergent genomic signatures of high-altitude adaptation among domestic mammals. Natl. Sci. Rev..

[33] Yan S., Pei F., Si J., Khan M.Y.A., Ou S., Yang Y., Zhao Z., Pauciullo A., Zhang Y. (2024). Gene co-expression network and differential expression analyses reveal key genes for weaning weight in Simmental-Holstein crossbred cattle. Anim. Biotechnol..

[34] Zhang S., Yao Z., Li X., Zhang Z., Liu X., Yang P., Chen N., Xia X., Lyu S., Shi Q. (2022). Assessing genomic diversity and signatures of selection in Pinan cattle using whole-genome sequencing data. BMC Genom..

[35] Tan Z., Jiang H. (2024). Molecular and cellular mechanisms of intramuscular fat development and growth in cattle. Int. J. Mol. Sci..

[36] Martins R., Machado P.C., Pinto L.F.B., Silva M.R., Schenkel F.S., Brito L.F., Pedrosa V.B. (2021). Genome-wide association study and pathway analysis for fat deposition traits innellorecattle raised in pasture-based systems. J. Anim. Breed. Genet..

[37] Silva-Vignato B., Cesar A.S.M., Afonso J., Moreira G.C.M., Poleti M.D., Petrini J., Garcia I.S., Clemente L.G., Mourao G.B., Regitano L.C.A. (2022). Integrative analysis between genome-wide association study and expression quantitative trait loci reveals bovine muscle gene expression regulatory polymorphisms associated with intramuscular fat and backfat thickness. Front. Genet..

[38] Cesar A.S., Regitano L.C., Mourão G.B., Tullio R.R., Lanna D.P., Nassu R.T., A Mudado M., Oliveira P.S., Nascimento M.L.D., Chaves A.S. (2014). Genome-wide association study for intramuscular fat deposition and composition in Nellore cattle. BMC Genet..

[39] Nasab S.E., Dashab G.R., Rokouei M., Roudbari Z., Sadkowski T. (2025). Unveiling Conserved Molecular Pathways of Intramuscular Fat Deposition and Shared Metabolic Processes in Semitendinosus Muscle of Hereford, Holstein, and Limousine Cattle via RNA-Seq Analysis. Genes.

[40] Zhou D., Wang Y., Yang R., Wang F., Zhao Z., Wang X., Xie L., Tian X., Wang G., Li B. (2022). The MyoD1 promoted muscle differentiation and generation by activating CCND2 in Guanling cattle. Animals.

[41] Flowers S., Hamblen H., Joel D.L.-G., Elzo M.A., Johnson D.D., Mateescu R.G. (2018). Fatty acid profile, mineral content, and palatability of beef from a multibreed Angus–Brahman population1. J. Anim. Sci..

[42] Zhang Y., Zhang J., Gong H., Cui L., Zhang W., Ma J., Chen C., Ai H., Xiao S., Huang L. (2019). Genetic correlation of fatty acid composition with growth, carcass, fat deposition and meat quality traits based on GWAS data in six pig populations. Meat Sci..

[43] Li R., Chen S., Li C., Xiao H., Costa V., Bhuiyan M.S.A., Baig M., Beja-Pereira A. (2022). Whole-Genome analysis deciphers population structure and genetic introgression among bovine species. Front. Genet..

[44] Neto L.R.P., Bunch R.J., Harrison B.E., Barendse W. (2012). Variation in the XKR4 gene was significantly associated with subcutaneous rump fat thickness in indicine and composite cattle. Anim. Genet..

[45] Cammack K.M., Thomas M.G., Enns R.M. (2009). Reproductive traits and their heritabilities in beef cattle. Prof. Anim. Sci..

[46] Sánchez J.M., Keogh K., Kelly A.K., Byrne C.J., Lonergan P., Kenny D.A. (2021). A high plane of nutrition during early life alters the hypothalamic transcriptome of heifer calves. Sci. Rep..

[47] Yang X., Wang Z., Chen Y., Ding H., Fang Y., Fang X., Liu H., Guo J., Zhao J., Wang J. (2024). ALKBH5 reduces BMP15 mRNA stability and regulates bovine puberty initiation through an m6A-dependent pathway. Int. J. Mol. Sci..

[48] Seminara S.B., Messager S., Chatzidaki E.E., Thresher R.R., Acierno J.S., Shagoury J.K., Bo-Abbas Y., Kuohung W., Schwinof K.M., Hendrick A.G. (2003). The GPR54 gene as a regulator of puberty. N. Engl. J. Med..

[49] Roa J., Tena-Sempere M. (2014). Connecting metabolism and reproduction: Roles of central energy sensors and key molecular mediators. Mol. Cell. Endocrinol..

[50] Hu B., Jin H., Shi Y., Yu H., Wu X., Wang S., Zhang K. (2024). Single-cell RNA-Seq reveals the earliest lineage specification and X chromosome dosage compensation in bovine preimplantation embryos. FASEB J..

[51] Hao Y., Hao S., Andersen-Nissen E., Iii W.M.M., Zheng S., Butler A., Lee M.J., Wilk A.J., Darby C., Zager M. (2021). Integrated analysis of multimodal single-cell data—ScienceDirect. Cell.

[52] Gasperini M., Hill A.J., José L.M.-F., Martin B., Kim S., Zhang M.D., Jackson D., Leith A., Schreiber J., Noble W.S. (2019). A Genome-wide framework for mapping gene regulation via cellular genetic screens. Cell.

[53] Kaya-Okur H.S., Wu S.J., Codomo C.A., Pledger E.S., Bryson T.D., Henikoff J.G., Ahmad K., Henikoff S. (2019). CUT&Tag for efficient epigenomic profiling of small samples and single cells. Nat. Commun..

[54] Buenrostro J.D., Giresi P.G., Zaba L.C., Chang H.Y., Greenleaf W.J. (2013). Transposition of native chromatin for fast and sensitive epigenomic profiling of open chromatin, DNA-binding proteins and nucleosome position. Nat. Methods.

[55] Cusanovich D.A., Daza R., Adey A., Pliner H.A., Christiansen L., Gunderson K.L., Steemers F.J., Trapnell C., Shendure J. (2015). Multiplex single-cell profiling of chromatin accessibility by combinatorial cellular indexing. Science.

[56] Chen L.L. (2020). The expanding regulatory mechanisms and cellular functions of circular RNAs. Nat. Rev. Mol. Cell Biol..

[57] Khan I.M., Liu H., Zhuang J., Khan N.M., Zhang D., Chen J., Xu T., Avalos L.F.C., Zhou X., Zhang Y. (2021). Circular RNA Expression and Regulation Profiling in Testicular Tissues of Immature and Mature Wandong Cattle. Front. Genet..

[58] Freitas P.H.F., Wang Y., Yan P., Oliveira H.R., Schenkel F.S., Zhang Y., Xu Q., Brito L.F. (2021). Genetic diversity and signatures of selection for thermal stress in cattle and other two Bos species adapted to divergent climatic conditions. Front. Genet..

[59] Colombi D., Perini F., Bettini S., Mastrangelo S., Abeni F., Conte G., Marletta D., Cassandro M., Bernabucci U., Ciampolini R. (2024). Genomic responses to climatic challenges in beef cattle: A review. Anim. Genet..

[60] Low W.Y., Tearle R., Liu R., Koren S., Rhie A., Bickhart D.M., Rosen B.D., Kronenberg Z.N., Kingan S.B., Tseng E. (2020). Haplotype-resolved genomes provide insights into structural variation and gene content in Angus and Brahman cattle. Nat. Commun..

[61] Tijjani A., Salim B., da Silva M.V.B., Eltahir H.A., Musa T.H., Marshall K., Hanotte O., Musa H.H. (2022). Genomic signatures for drylands adaptation at gene-rich regions in African zebu cattle. Genomics.

[62] Ramírez-Ayala L.C., Rocha D., Ramos-Onsins S.E., Leno-Colorado J., Charles M., Bouchez O., Rodríguez-Valera Y., Pérez-Enciso M., Ramayo-Caldas Y. (2021). Whole-genome sequencing reveals insights into the adaptation of French Charolais cattle to Cuban tropical conditions. Genet. Sel. Evol..

[63] Xu L., Yang L., Zhu B., Zhang W., Wang Z., Chen Y., Zhang L., Gao X., Gao H., Liu G.E. (2019). Genome-wide scan reveals genetic divergence and diverse adaptive selection in Chinese local cattle. BMC Genom..

[64] Knap P.W., Doeschl-Wilson A. (2020). Why breed disease-resilient livestock, and how?. Genet. Sel. Evol..

[65] Prat-Benhamou A., Meuwissen M., Slijper T., Bernués A., Gaspar-García P., Lizarralde J., Mancilla-Leytón J., Mandaluniz N., Mena Y., Soriano B. (2025). A practical approach to assess the resilience attributes of livestock farms. Animal.

[66] Yan C.-L., Lin J., Huang Y.-Y., Gao Q.-S., Piao Z.-Y., Yuan S.-L., Chen L., Ren X., Ye R.-C., Dong M. (2022). Population genomics reveals that natural variation in PRDM16 contributes to cold tolerance in domestic cattle. Zool. Res..

[67] Ghoreishifar S.M., Eriksson S., Johansson A.M., Khansefid M., Moghaddaszadeh-Ahrabi S., Parna N., Davoudi P., Javanmard A. (2020). Signatures of selection reveal candidate genes involved in economic traits and cold acclimation in five Swedish cattle breeds. Genet. Sel. Evol..

[68] Huang K., Li Z., Zhong D., Yang Y., Yan X., Feng T., Wang X., Zhang L., Shen X., Chen M. (2023). A circular RNA generated from Nebulin (NEB) gene splicing promotes skeletal muscle myogenesis in cattle as detected by a multi-Omics approach. Adv. Sci..

[69] Cao Y., Jia P., Wu Z., Huang M., Chen S., Zhang J., Huang B., Lei C. (2020). A novel SNP of MYO1A gene associated with heat-tolerance in Chinese cattle. Anim. Biotechnol..

[70] Ma X., Liu Y., Sun L., Hanif Q., Qu K., Liu J., Zhang J., Huang B., Lei C. (2021). A novel SNP of TECPR2 gene associated with heat tolerance in Chinese cattle. Anim. Biotechnol..

[71] Zeng L., Chen N., Ning Q., Yao Y., Chen H., Dang R., Zhang H., Lei C. (2018). PRLH and SOD1 gene variations associated with heat tolerance in Chinese cattle. Anim. Genet..

[72] Mei C., Wang H., Liao Q., Wang L., Cheng G., Wang H., Zhao C., Zhao S., Song J., Guang X. (2018). Genetic architecture and selection of Chinese cattle revealed by whole genome resequencing. Mol. Biol. Evol..

[73] Wang S., Liu J., Zhao W., Wang G., Gao S. (2021). Selection of candidate genes for differences in fat metabolism between cattle subcutaneous and perirenal adipose tissue based on RNA-seq. Anim. Biotechnol..

[74] Gim G.-M., Uhm K.-H., Kwon D.-H., Kim M.-J., Jung D.-J., Kim D.-H., Yi J.-K., Ha J.-J., Yum S.-Y., Son W.-J. (2022). Germline transmission of MSTN knockout cattle via CRISPR-Cas9. Theriogenology.

[75] Messmer T., Dohmen R.G.J., Schaeken L., Melzener L., Hueber R., Godec M., Didoss C., Post M.J., Flack J.E. (2023). Single-cell analysis of bovine muscle-derived cell types for cultured meat production. Front. Nutr..

[76] Bai F., Cai Y., Qi M., Liang C., Pan L., Liu Y., Feng Y., Cao X., Yang Q., Ren G. (2025). LCORL and STC2 variants increase body size and growth rate in cattle and other animals. Genom. Proteom. Bioinform..

[77] Tan X., Zhao R., Chen J., Yan Z., Sui X., Li H., Li Q., Du X., Liu Y., Yao S. (2025). Integrative transcriptomic, proteomic and metabolomic analyses yields insights into muscle fiber type in cattle. Food Chem..

[78] Song Y., Zhang J., Jiang C., Song X., Chen X., Raza S.H.A., Pant S.D., Ma Y., Zan L., Wei D. (2025). Vitamin A mediates FABP4 to regulate intramuscular fat production: A new target and strategy for optimizing beef quality. BMC Genom..

[79] Liang M., An B., Deng T., Du L., Li K., Cao S., Du Y., Xu L., Zhang L., Gao X. (2023). Incorporating genome-wide and transcriptome-wide association studies to identify genetic elements of longissimus dorsi muscle in Huaxi cattle. Front. Genet..

[80] Imai K., Keele L., Tingley D. (2010). A general approach to causal mediation analysis. Psychol. Methods.

[81] Marrella M.A., Biase F.H. (2023). A multi-omics analysis identifies molecular features associated with fertility in heifers (Bos taurus). Sci. Rep..

[82] Chen N., Cai Y., Chen Q., Li R., Wang K., Huang Y., Hu S., Huang S., Zhang H., Zheng Z. (2018). Whole-genome resequencing reveals world-wide ancestry and adaptive introgression events of domesticated cattle in East Asia. Nat. Commun..

[83] Wang L., Gao P., Li C., Liu Q., Yao Z., Li Y., Zhang X., Sun J., Simintiras C., Welborn M. (2023). A single-cell atlas of bovine skeletal muscle reveals mechanisms regulating intramuscular adipogenesis and fibrogenesis. J. Cachexia Sarcopenia Muscle.

[84] Jang J., Kim K., Lee Y.H., Kim H. (2021). Population differentiated copy number variation of Bos taurus, Bos indicus and their African hybrids. BMC Genom..

[85] Salehian-Dehkordi H., Xu Y.X., Xu S.S., Li X., Luo L.Y., Liu Y.J., Wang D.F., Cao Y.H., Shen M., Gao L. (2021). Genome-wide detection of copy number variations and their association with distinct phenotypes in the world’s sheep. Front. Genet..

[86] Fodor I., Spoelstra M., Calus M.P.L., Kamphuis C. (2023). A systematic review of genotype-by-climate interaction studies in cattle, pigs, and chicken. Front. Anim. Sci..

[87] Filho I.C., Campos G.S., Lourenco D., Schenkel F.S., da Silva D.A., Silva T.L., Teixeira C.S., Fonseca L.F.S., Júnior G.A.F., de Albuquerque L.G. (2025). Genotype by environment interaction for productive and reproductive traits in beef cattle using imputed whole genome sequence. J. Appl. Genet..

[88] Sartori C., Tiezzi F., Guzzo N., Mancin E., Tuliozi B., Mantovani R. (2022). Genotype by environment interaction and selection response for milk yield traits and conformation in a local cattle breed using a reaction norm approach. Animals.

[89] Jarquin D., Crossa J., Lacaze X., Du Cheyron P., Daucourt J., Lorgeou J., Piraux F., Guerreiro L., Perez P., Calus M. (2014). A reaction norm model for genomic selection using high-dimensional genomic and environmental data. Theor. Appl. Genet..

[90] MacLeod I.M., Bowman P.J., Vander Jagt C.J., Haile-Mariam M., Kemper K.E., Chamberlain A.J., Schrooten C., Hayes B.J., Goddard M.E. (2016). Exploiting biological priors and sequence variants enhances QTL discovery and genomic prediction of complex traits. BMC Genom..

[91] Teissier M., Larroque H., Robert-Granie C. (2018). Weighted single-step genomic BLUP improves accuracy of genomic breeding values for protein content in French dairy goats: A quantitative trait influenced by a major gene. Genet. Sel. Evol..

[92] Raymond B., Yengo L., Costilla R., Schrooten C., Bouwman A.C., Hayes B.J., Veerkamp R.F., Visscher P.M. (2020). Using prior information from humans to prioritize genes and gene-associated variants for complex traits in livestock. PLoS Genet..

[93] Wiggans G.R., Cole J.B., Hubbard S.M., Sonstegard T.S. (2016). Genomic selection in dairy cattle: The USDA experience. Annu. Rev. Anim. Biosci..

[94] Yu H., Wang J., Zhang K., Cheng G., Mei C., Zan L. (2023). Integrated multi-omics analysis reveals variation in intramuscular fat among muscle locations of Qinchuan cattle. BMC Genom..

[95] Libbrecht M.W., Noble W.S. (2015). Machine learning applications in genetics and genomics. Nat. Rev. Genet..

[96] Gustavo D.L.C., Gianola D., Allison D.B. (2010). Predicting genetic predisposition in humans: The promise of whole-genome markers. Nat. Rev. Genet..

[97] Zhang J., Che Y., Liu R., Wang Z., Liu W. (2025). Deep learning–driven multi-omics analysis: Enhancing cancer diagnostics and therapeutics. Brief. Bioinform..

[98] Montesinos-Lopez O.A., Montesinos-Lopez A., Perez-Rodriguez P., Barron-Lopez J.A., Martini J.W.R., Fajardo-Flores S.B., Gaytan-Lugo L.S., Santana-Mancilla P.C., Crossa J. (2021). A review of deep learning applications for genomic selection. BMC Genom..

[99] Jubair S., Domaratzki M. (2022). Crop genomic selection with deep learning and environmental data: A survey. Front. Artif. Intell..

